# PRECIS-2 analysis of pragmatic acupuncture trials: a systematic review

**DOI:** 10.1186/s12906-024-04473-7

**Published:** 2024-05-03

**Authors:** Jinwoong Lim, Hyeonhoon Lee, Yong-Suk Kim

**Affiliations:** 1 Department of Acupuncture and Moxibustion, Wonkwang University Gwangju Korean Medicine Hospital, Gwangju, Republic of Korea; 2 Department of Anesthesiology and Pain Medicine, Seoul National University Hospital, Seoul, Republic of Korea; 3 Biomedical Research Institute, Seoul National University Hospital, Seoul, Republic of Korea; 4 Department of Acupuncture and Moxibustion Medicine, College of Korean Medicine, Kyung Hee University, 23 Kyunghee Dae-Ro, Dongdaemun-Gu, Seoul, 02447 Republic of Korea

**Keywords:** Pragmatic trial, Acupuncture, PRECIS-2

## Abstract

**Background:**

Pragmatic acupuncture trials (PATs) are a research tool for assessing the effectiveness of acupuncture treatments in a real-world setting. This study aimed to provide a comprehensive methodological analysis of PATs using the PRECIS-2(PRagmatic Explanatory Continuum Indicator Summary-2) tool to determine their pragmatism.

**Methods:**

The MEDLINE, EMBASE, Cochrane Central Register for Controlled Trials, CINAHL, Allied and Complementary Medicine Database, China National Knowledge Infrastructure, VIP, WANFANG, Taiwan Periodical Literature Database, KoreaMed, KMbase, Research Information Service System, Oriental Medicine Advanced Searching Integrated System, CiNii and ClinicalTrials.gov were searched. The search included randomised controlled trials (RCTs) and protocols of RCTs that investigated all types of acupuncture and used self-declared pragmatic design. Two authors independently collected the basic information and characteristics of the studies and assessed their pragmatism using the nine PRECIS-2 domains and the additional domain of control.

**Results:**

A total of 93 studies were included. The means of eligibility, recruitment, organisation, primary outcome, primary analysis, and control domains were statistically larger than three and were shown to be pragmatic. The means of setting, flexibility:delivery, and follow-up domains were not greater than three and were shown to be non-pragmatic. For flexibility:adherence domain was inappropriate for assessment owing to insufficient information in the studies.

**Conclusions:**

PATs were pragmatic in the domain of eligibility, recruitment, organisation, primary outcome, primary analysis, and control and were not pragmatic in the domain of setting, flexibility:delivery, and follow-up. Future PATs need to strengthen the pragmatism in the setting, flexibility:delivery, and follow-up domains and to describe the flexibility:adherence domain in more detail.

**Trial registration:**

CRD42021236975.

**Supplementary Information:**

The online version contains supplementary material available at 10.1186/s12906-024-04473-7.

## Background

Acupuncture has been practiced for thousands of years with acupuncture research beginning in the eighteenth century [1]. However, owing to the large disjunction between the histories of clinical practice and clinical trials, acupuncture trials have continuously been controversial regarding the disharmony between real-world practice and experimental interpretation [2]. For instance, acupuncture’s placebo effect has thus far not been clearly described, yet it is a crucial factor for identifying the exact efficacy of this treatment in randomised controlled trials (RCTs) [3].

The term pragmatic trial refers to a trial conducted in a realistic simulated practice setting that focuses on the effectiveness of treatments as opposed to an explanatory trial [4]. The trial method has been proposed as a superior way to assess the clinical benefit of acupuncture [5–8]. Recently, some acupuncture trials have been conducted with a pragmatic-oriented direction; however, there are unanswered questions regarding whether these trials have been conducted with real-world settings or have provided an accurate assessment of acupuncture treatment.

PRagmatic Explanatory Continuum Indicator Summary-2 (PRECIS-2) is a tool for designing pragmatic trials and is developed by Loudon et al. [9]. It includes the following nine domains: eligibility, recruitment, setting, organisation, flexibility:delivery, flexibility:adherence, follow-up, primary outcome, and primary analysis. Zwarenstein et al. have reported the retrospective use of the PRECIS-2 tool in published RCTs [10]. Since acupuncture trials must employ a pragmatic design to show the effectiveness of acupuncture, it is necessary to analyse how pragmatically they are designed before assessing acupuncture’s effectiveness in a pragmatic setting. However, no comprehensive methodological analysis of the pragmatic acupuncture trials (PATs) has yet been reported. Therefore, we conducted a systematic review of PATs and assessed the pragmatism of the trials using the PRECIS-2 tool to inform a proper direction for future PATs.

## Methods

The present study has been registered in the PROSPERO (CRD42021236975); however, the protocol has been revised before the publication and some parts of the methods have been altered from the first registration. The method of the present study was based on the previously published protocol [11].

### Literature search strategy

We searched fifteen electronic databases (MEDLINE, EMBASE, the Cochrane Central Register for Controlled Trials, CINAHL, Allied and Complementary Medicine Database, China National Knowledge Infrastructure, VIP, WANFANG, Taiwan Periodical Literature Database, KoreaMed, KMbase, Research Information Service System, Oriental Medicine Advanced Searching Integrated System, CiNii and ClinicalTrials.gov for registered trials). The search terms for the databases are provided in the previous protocol [11]. Appropriate articles were manually retrieved when necessary. This study was conducted by the Preferred Reporting Items for Systematic Reviews and Meta-analyses reporting guidelines [12]. (see Additional file 1).

### Eligibility criteria

We reviewed RCTs and RCT protocols published before March 2022 that investigated or planned to investigate any type of acupuncture including manual acupuncture, electroacupuncture, microsystem acupuncture such as auricular acupuncture, and acupoint acupressure. The inclusion criteria were as follows: 1) RCTs and RCT protocols that mentioned pragmatic trial design or pragmatic treatment in the title, abstract, or manuscript, and 2) RCTs and RCT protocols for interventions that included acupuncture treatment. The exclusion criteria were as follows: 1) protocols of published RCTs, 2) secondary analyses of published RCTs, and 3) studies that used the word ‘pragmatic’ not in a methodological manner. Two researchers independently screened the articles based on the inclusion and exclusion criteria.

### Study selection

After excluding duplicates, two independent researchers selected the studies based on the criteria. A discussion was held with the third party if there was inconsistency.

### Data extraction and analysis

Two independent researchers extracted the bibliographical and basic information from the selected studies. To assess the pragmatism of the included studies, they scored the nine domains of the PRECIS-2 tool for each study and experimentally added the additional domain of control. This domain is currently not included in the PRECIS-2 tool; however, Zwarenstein et al. recommend a control-related domain to clarify whether a control group has been pragmatically designed when retrospectively assessing trials [10]. Scoring criteria were based on Loudon et al. [9] and the exact criteria suitable for the characteristics of acupuncture trials were further discussed. The control domain was assigned a score of five when the control group was a usual care group without any restrictions on treatments and scored one when the control group was a sham-controlled group as Zwarenstein et al. suggested [10]. We attempted to find any additional available information for protocols or other related articles of included RCTs to score the domains; however, if there was insufficient information, the scores were left blank as suggested by Loudon et al. [9]. Two researchers independently scored the studies and discussed the scores. If there was inconsistency, a discussion was held with a third researcher. If inconsistency remained following the discussion, the mean scores of the first two researchers were used. To obtain consistency, a conference for understanding the criteria of the PRECIS-2 tool and the characteristics of the acupuncture trial and discussing the score was held once a week for six months. First, 10% of the included studies were scored, and three authors established the detailed criteria for acupuncture trials. Then after scoring all the studies, two reviewers independently re-checked the score and rationale and finally confirmed the scores. When there were discrepancies regarding data extractions and analysis, we engaged a third reviewer to resolve the issues ensuring our results were accurate and reliable. All the authors were specialists in acupuncture and moxibustion certified by the Ministry of Health and Welfare of the Republic of Korea and have more than five years of experience in acupuncture trials and clinical practice.

### Statistical analysis

The descriptive statistics of the PRECIS-2 scores were investigated with mean and standard deviation calculations. Based on the bibliographical characteristics, the scores were assessed by subgroup analysis. According to Loudon et al., [9] for the domains of flexibility:delivery, flexibility:adherence, and control, if there were more than two groups, each group could be scored separately. However, when it came to statistical analysis, we used the score of the acupuncture-related group, and if all groups were related to acupuncture, we used the highest score to reflect the potential pragmatism of the trial. A one-sample t-test was used to investigate whether the score was greater than three (equally pragmatic and explanatory), and *p* < 0.05 was considered statistically significant.

## Results

**Fig. 1:**
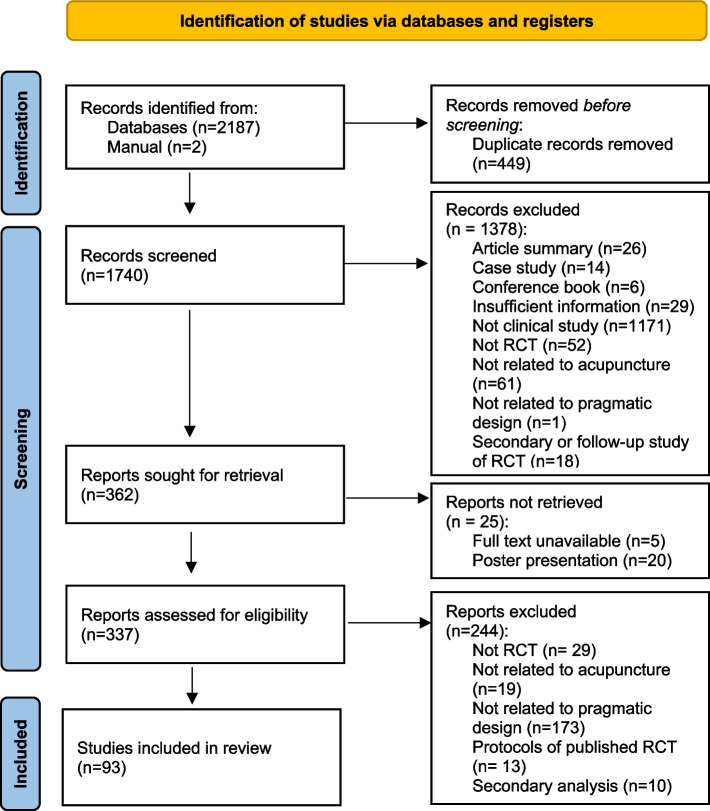
Flow chart of the study

In total, 1,740 studies were found after searching and excluding duplicates. Based on the titles and abstracts, 1,647 studies were excluded, and 93 studies were finally included in the review. Two studies [13, 14] were considered to be the same trial; however, they reported different outcomes. Hence, we reviewed both articles. The flow chart [12] of this study is shown in Fig. 1.

Sixteen studies were published until 2009 when PRECIS was first presented, [15] and 27 more were published between 2010 and 2015 when PRECIS-2 was presented. Thirty-eight studies in European countries (14 in the UK; 10 in Germany; 3 in Norway; 2 each in Denmark, Italy, Spain, and Sweden; 1 each in France, Greece, and the Netherlands), 35 studies in East Asian countries (21 in China; 9 in Korea; 3 in Japan; 1 each in Singapore and Hong Kong), 9 studies in the United States, 5 studies in Australia, 3 studies in Brazil, and 1 study each in Canada, Israel, and New Zealand was conducted. Three of the studies were written in Korean, Chinese, and German, and one was written in Japanese. Twenty-seven were protocol articles, 18 studies were pilot or feasibility trials, and one study was an interim analysis. The bibliographic characteristics of these studies are summarised in the Additional file 1.

### PRECIS-2 scores

#### Overall results

**Fig. 2:**
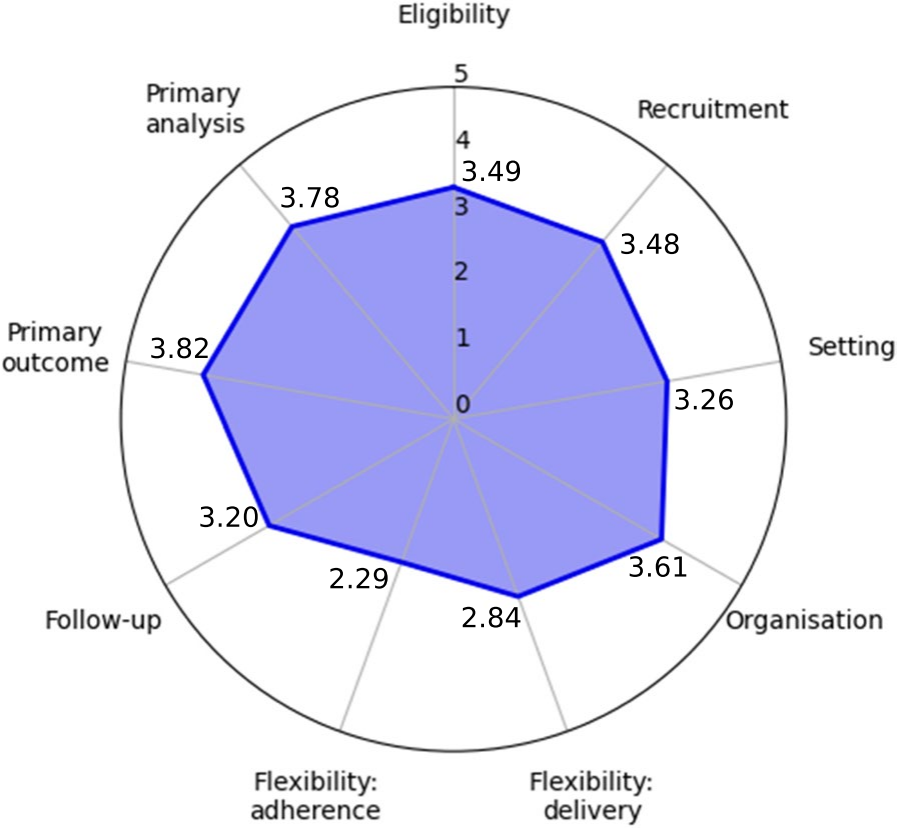
PRECIS-2 score of included studies

The mean ± standard deviation (p-value) values of 10 domains (eligibility, recruitment, setting, organisation, flexibility:delivery, flexibility:adherence, follow-up, primary outcome, primary analysis, and control) were 3.49 ± 1.08 (*p* < 0.01), 3.48 ± 1.47 (*p* < 0.01), 3.26 ± 1.37(*p* = 0.06), 3.61 ± 1.43 (*p* < 0.01), 2.84 ± 1.50 (*p* = 0.83), 2.29 ± 0.83 (*p* = 0.99), 3.20 ± 1.24 (*p* = 0.07), 3.82 ± 1.22 (*p* < 0.01), 3.78 ± 1.73 (*p* < 0.01) and 3.76 ± 1.18 (*p* < 0.01) respectively (Fig. 2). The mean ± standard deviation (*p*-value) of the average score of nine domains in each study was 3.41 ± 0.64 (*p* < 0.01). The key factors of scoring in each RCT were summarised in Table 1. Individual scores of the studies and the scores of nine domains divided by subgroups [publication years before 2009, between 2010 and 2015, and after 2015; countries where five or more studies were reported (China, the United Kingdom, Germany, Korea, the United States of America, and Australia); type of study (pilot or feasibility trial and original RCT)] are shown in the Additional file 2.

**Table 1:** Key pragmatic and explanatory factors used to score the PRECIS-2 of the included RCTs

Score	1	2	3	4	5
Eligibility	Required extensive data (more than laboratory results and questionnaire scores)	Required laboratory results, questionnaire scores, daily diaries before enrolment, and excluded common conditions and medication	Required laboratory results, setting a specific cut off, or excluded common comorbidities, medication	Strict criteria such as excluding common diseases or comorbidities	Loose criteria without specific limitations
Recruitment	Recruited participants from one specific unit, used massive advertisements, or compensated participants	Used advertisements in centres or newspapers or mailed to extensive groups of people	Recruited participants as in the usual setting and additionally advertised the trial	Advertised to patients who visited clinics or searched specialised hospital registries	Only through referrals in the usual setting
Setting	Single-centre trials of specialised or tertiary hospitals on diseases or conditions which are usually treated by GPs	N/A	Conducted in single centre	Conducted in two GPs or two specialised hospitals	Conducted in more than two GPs or hospitals
Organisation	Required highly experienced and trained practitioners with an academic degree and additional professionals	Required extensive additional training, various kinds of professionals, or an academic degree	Required moderate additional training	Acupuncture practitioners with 10 years of experience	Required a minimum qualification
Flexibility:delivery	Standardised acupuncture treatments within a strict discipline	Standardised and defined strictly as treatment sessions and cointervention	Semi-individualised (flexible within certain standard treatment procedures)	Generally individualised but minimally fixed number of sessions or co-intervention	Highly individualised and flexible treatment
Flexibility:adherence	Compensated participants at each follow-up visit	Excluded poor compliance or measured various outcomes to promote adherence	Fixed minimum treatment sessions prior to inclusion	N/A	Allowed non-attendance or sent messages for upcoming sessions as in the usual care
Follow-up	Intense follow-ups in terms of frequency and duration, and extensive outcome measurements	Intense follow-ups in terms of frequency and duration and quite extensive outcome measurements	Quite intense follow-ups in terms of frequency and duration	Follow-ups not extensive in terms of frequency and duration	No follow-ups after the end of the treatment session or medical records were used
Primary outcome	Used various outcomes as primary outcomes and required additional professionals and equipment	Unrelated outcome measurements or outcome measurements requiring intense data collection	Questionnaires that were less commonly used in the usual care	Questionnaires related to the disease or condition	Pragmatic measurements such as the visual analogue scale or objective outcomes using medical records
Primary analysis	Per-protocol analysis or excluded missing data and poor compliance	N/A	N/A	Used a full analysis set or modified intention-to-treat	Analysed all the participants at randomisation
Control	Used sham-acupuncture treatment	Physical touch that tended to function as sham acupuncture	Fixed treatments available in usual care	Restriction placed on usual care	Used usual care without specific restrictions

*PRECIS-2* PRagmatic Explanatory Continuum Indicator Summary-2, *RCT* Randomised controlled trial, *GP* General practice, *N/A* Not available

#### Eligibility

We assessed whether inclusion or exclusion criteria unnecessarily narrowed the number of possible participants compared to usual care. Contraindications to acupuncture and usual diagnostic criteria were not considered during scoring.

Fifteen studies that stated loose criteria without specific limitations were scored as five. Thirty-six studies with strict criteria, such as excluding common diseases or comorbidities, were considered pragmatic (scored as four). One study was scored as 3.5 since the study included patients with rather severe conditions. Twenty-one studies were scored as three, and the reasons for this included requiring laboratory results, setting a specific cut-off, or excluding common comorbidities, medication, low education level, and other diseases that could have affected the results of the intervention. One study was scored as 2.5 since it required specific inclusion criteria and excluded patients with common conditions and acupuncture experience. Twelve studies were scored as two, and these studies stated stricter criteria that required laboratory results, questionnaire scores, and daily diaries before enrolment, and excluded common conditions and medication that could have affected the results of the intervention. One study that required endoscopic results and excluded common comorbidities was scored as 1.5. Four studies were scored as one as they required extensive data before enrolment and exclusion of various conditions and diseases. Two studies did not properly state the inclusion/exclusion criteria, and thus the score was left blank.

#### Recruitment

In the recruitment domain, we assessed whether additional strategies rather than the usual setting were used as part of the recruitment method.

Twenty-six studies were scored as five as they recruited participants only through referrals. They contacted eligible persons who visited a clinic or were admitted or mailed eligible patients after searching a local database or registry (achievable in the usual setting) [9]. Eight studies were scored as four since they advertised the trial only to patients who visited clinics or searched specialised hospital registries. Five studies were scored as three because they recruited participants as in the usual setting and additionally advertised the trial. One study that used massive advertisements and contacted possible patients through an outpatient clinic scored as 2.5. Nineteen studies were scored as two because they used advertisements in centres or newspapers or mailed to extensive groups of people who may have been ineligible from a database. Five studies were scored as one as some recruited participants from one specific unit, used massive advertisements, or compensated participants.Twenty-nine studies did not properly report their recruitment methods, and the score was left blank.

#### Setting

We assessed the appropriateness of study centres for managing patient conditions and research on the delivery of acupuncture or other interventions as well as the number of study centres.

Nineteen studies were scored as five as they were conducted in more than two general practices (GPs) or hospitals according to the disease or condition they investigated. Six studies were scored as four because they included two GPs or two specialised hospitals appropriately in accordance with participant conditions. Thirty-two studies were scored as three as they had conducted single-centre trials appropriately in accordance with participant conditions. Thirteen studies were scored as one because they conducted single-centre trials of specialised or tertiary hospitals on diseases or conditions which are usually treated by GPs. Twenty-three studies were scored as blank since they either failed to mention where the study was conducted and treatment delivered or reported that they conducted a multicentre trial, but the types of centres or clinics were not clearly identified.

#### Organisation

We assessed the experience level of the acupuncture practitioners and the level of equipments used in the included studies.

Thirty-seven studies were scored as five as they required a minimum qualification that was required to practise acupuncture in each country without additional training. Six studies were scored as four because they included acupuncture practitioners with 10 years of experience. Twelve studies were scored as three since they required moderate additional training to perform acupuncture. One study was scored as 2.5 as it included two types of professionals without extensive training. Twenty studies were scored as two since they required extensive additional training, various kinds of professionals, or an academic degree. Five studies were scored as one as they required highly experienced and trained practitioners with an academic degree and additional professionals. Twelve studies either did not report or insufficiently reported the practitioner’s information and thus were scored as blank.

#### Flexibility: delivery

We also assessed how much acupuncture treatments were individualised or based on the discretion of practitioners.

Seventeen studies were scored as five as acupuncture point selection and treatment were highly individualised and flexible at the discretion of practitioners. One study was scored as 4.5 since the delivery was highly flexible, but a protocol was suggested. Fifteen studies were scored as four as acupuncture prescriptions were generally individualised and flexible based on the practitioner’s discretion; however, other treatment regimens such as the number of treatment sessions or co-intervention were minimally fixed. Fifteen studies were scored as three since they showed semi-individualised (flexible within certain standard treatment procedures) acupuncture treatments, fixed treatment sessions, and cointerventions. Sixteen studies were scored as two as their acupuncture treatments were standardised and defined strictly as treatment sessions and cointervention with adherence management of practitioners. Twenty-four studies were scored as one since they thoroughly investigated standardised acupuncture treatments within a strict discipline. Five studies did not report sufficient information on the delivery of the intervention and thus were scored blank.

#### Flexibility: adherence

Any specific method used to manage the adherence of participants to intervention was assessed.

Two studies were scored as four since they attempted to maintain adherence as in usual care by allowing non-attendance of participants or sending messages for upcoming sessions. One study was scored as three because the study fixed minimum treatment sessions prior to inclusion. Ten studies were scored as two because they excluded poor compliance from trials or measured various outcomes to promote adherence. One study was scored as one as it compensated participants at each follow-up visit. Seventy-nine studies either did not sufficiently report any methods regarding adherence or were not applicable.

#### Follow-up

The frequency and duration of follow-ups, additional data collection, or any other methods regarding follow-up management were also measured. Typically, acupuncture treatments consist of several sessions, and therefore follow-up outcome assessments during treatment sessions were considered pragmatic unless they were more extensive than usual care.

Thirteen studies were scored as five since either participants were not followed up after the end of the treatment session or medical records were used for follow-up assessments. Thirty studies were scored as four because they carried out follow-up assessments, but they were not extensive in terms of frequency and duration. Twenty-three studies were scored as three because their follow-up assessments were considered quite intense in terms of frequency and duration. Fourteen studies were scored as two since the follow-ups were intense as compared with usual care and quite intense with extensive outcome measurements or excessive reminders used. Twelve studies were scored as one since the follow-ups were intense in terms of frequency and duration, and extensive outcome measurements were collected with participants either individually contacted to turn in outcome measurements or compensated on each follow-up and at the end of the study. One study did not properly state the follow-up strategy and was scored as a blank.

#### Primary outcome

We additionally measured whether the primary outcome was patient-centred and available in usual care. If there were many outcomes like in the pilot study, the outcome used to calculate the sample size was considered the primary outcome.

Thirty-three studies used pragmatic measurements such as the visual analogue scale or objective outcomes using medical records and were scored as five. Thirty studies were scored as four as most of them used questionnaires related to the disease or condition, and this was regarded as pragmatic. Five studies were scored as three because they employed questionnaires that were less commonly used in usual care. Two studies were scored as 2.5 since they used questionnaires that were unrelated to the disease or condition. Sixteen studies were scored as two because they used unrelated outcome measurements or outcome measurements requiring intense data collection such as daily diaries or additionally trained assessors. Three studies were scored as one because they used various outcomes as primary outcomes and required additional professionals and equipment. Four studies did not determine the primary outcome and measured various outcomes without sample calculation, and the score for these was left blank.

#### Primary analysis

We also assessed the statistical methods such as the intention-to-treat (ITT) and per-protocol analysis.

Fifty studies analysed all the participants at randomisation and were scored as five. Twelve studies were scored as four since they used a full analysis set or modified ITT (for instance, including participants who received at least one treatment session) even though they used ITT analysis. Twenty-three studies were scored as one since they used the per-protocol analysis or excluded missing data and poor compliance despite the fact that they used ITT analysis. Eight studies did not report the analysis methods, and these scores were left blank.

#### Control

We also measured which interventions were used in control groups. Studies that provided usual care without any discipline to their control groups were scored as five; thus, 27 studies that used usual care without specific restrictions as the control group were scored as five. Eighteen studies were scored as four since there was a restriction placed on usual care in the control group. Twenty-six studies were scored as three since they used fixed treatments available in usual care as the control group. One study was scored as two since the study used physical touch in the control group that tended to function as sham acupuncture. Six studies used sham-acupuncture treatment in the control groups and were scored as one. Fifteen studies that did either not report the extent to which usual care or sham treatment was applied in the control group or had no available control group (for instance, all groups were using acupuncture treatment for experimental purposes) were left blank in this domain.

## Discussion

As Dal-Ré et al. have previously reported, some self-labelled pragmatic trials have not been properly pragmatically designed, [16] and this tendency is observed among PATs. In this study, we assessed the pragmatism of PATs and aimed to suggest an appropriate direction for future PATs.

As Ian Ford et al. have stated, few trials are pragmatic in all domains of the PRECIS-2 tool, and most trials show pragmatism in certain domains [17]. In the same manner, acupuncture trials may be designed pragmatically in one or two domains; however, self-labelled PATs need to strengthen their pragmatic methods and report them in detail for each domain of the PRECIS-2 tool with rationale considering the results of this systematic review.

The eligibility, recruitment, organisation, primary outcome, and primary analysis domains were shown to be pragmatic (with a mean value greater than three) for the studies in this analysis. These results are encouraging as acupuncture intervention is commonly used to treat daily life conditions and needs no additional training or qualifications to the institutional qualification needed in each country. For these domains, it would be desirable for researchers to embed a real-world setting in future PATs.

Setting, flexibility:delivery, and follow-up domains were not shown to be pragmatic. Several issues need to be addressed for these domains. For setting, the factors reducing the score in this domain included the conduction of some trials in hospitals or specialised centres when the diseases or conditions are usually treated in GPs in real life. Delivering acupuncture itself usually does not require a high degree of a clinical setting. So the location in which the condition or disease that the trial aims to investigate is treated in usual care settings needs to be considered more in PATs. Additionally, as Zwarenstein et al. [10] argued, single centres were not considered completely explanatory in this review; however, the number of centres needs to be expanded to apply the results generally in real-world practice settings. For flexibility:delivery, this domain may be considered the most important factor in PATs; however, the mean was below three and did not show pragmatism in the t-test. The main reasons for this were strict acupuncture delivery protocols that limited the acupuncture points, treatment sessions, cointerventions, and interactions between practitioners and patients. Of course, the specific acupuncture point and treatment sessions can be suggested through cumulative evidence such as the point pericardium 6 for postoperative nausea and vomiting [18]. However, a primary characteristic of acupuncture is individualised and complex treatment [19, 20] and is commonly emphasised in the usual care setting. Even in the Standards for Reporting Interventions in Clinical Trials of Acupuncture (STRICTA), [21] descriptions of individualised and pragmatic acupuncture protocols and a certain degree of flexibility are allowed. Future researchers conducting PATs need to consider this domain. The follow-up domain was not pragmatic; the principal reason for this was the extensive data collection at follow-up periods as well as the frequency and duration of follow-ups. As acupuncture treatment usually deals with the usual day-to-day conditions, follow-ups need to be simplified. Although the primary outcomes were pragmatic in the studies, various secondary outcomes occasionally requiring special equipment and additional assessors reduced the score in this domain. The primary purpose of clinical trials is significant; however, extensive outcome measurements and follow-up procedures could potentially compromise the practical applicability of acupuncture trials in real-world settings. The flexibility:adherence domain could not be appropriately assessed in this review because of insufficient information in the studies. Along with the recruitment domain for which 31.2% of the studies were left blank, the rate of the score ‘blank’ indicating a lack of sufficient information provided in the studies to assess the domain was 84.9% and relatively higher than other domains, and this tendency was shown in a previous study assessing integrative medicine research [22]. In the real-world setting, methods such as sending messages or making phone calls for upcoming appointments may plausibly be used; however, attendance would not be compulsorily forced. Future PATs should explore this issue in more detail.

The control groups in the studies were experimentally scored and shown to be pragmatic. This serves as evidence that the control group is a feature in pragmatic trials; conversely, as Zwarenstein et al.[10] argued, the placebo-controlled group could be pragmatic in some circumstances, so that the rationale for choosing the sham-controlled group as a control group needs to be further described in future pragmatic acupuncture studies.

We have summarised overall score of the PRECIS-2 domain in countries where more than 5 studies were published, (Additional file 2) and the average scores were 2.89, 2.84, 3.75, 3.31, 3.66 and 3.61 for China, Korea, UK, USA, Germany and Australia, respectively. Surprisingly, RCTs conducted in two East Asian countries that have used acupuncture for thousand years tended to be less pragmatic, and the tendency needs further investigations in future studies. For the analysis based on the publication year, (Additional file 2) the average scores were 3.73, 3.24 and 3.24 for before 2009, between 2010–2015, and after 2015 respectively. The more recent studies showed less pragmatic findings; however, the scores indicate minimal differences across the year.

The strengths of this systematic review are that it was, to the best of our knowledge, the first study to conduct a comprehensive review of PATs and assess the pragmatism of the trials. In particular, we highlighted the insufficient features of the trials which must be further improved and described in future PATs to help decision-makers such as doctors, patients, and policymakers to efficiently utilise the results of future PATs.

The limitations of the review are as follows: 1) Assessment was based on published articles. So if trials were conducted more pragmatically or differently from the articles, the score was able to be changed. This issue will be improved when future articles report more detailed pragmatic methods as well as how real trials are performed according to the extended version of the Consolidated Standards of Reporting Trials statement[23] and in acupuncture the Standards for Reporting Interventions in Clinical Trials of Acupuncture statement [21]; 2) When scoring PRECIS-2 domains, it is unreasonable to recognise that the one-point difference in the domains reflects an exact one-degree difference in pragmatism of the trial. Since this study quantitatively utilised the score from the qualitative information from the studies for descriptive analysis and understanding the status of PATs and used statistical analysis as little as possible, the pragmatism of the individual studies should be interpreted with caution.

## Conclusions

Eligibility, recruitment, organisation, primary outcome, and primary analysis domains of the PRECIS-2 tool were shown to be quite pragmatic in PATs, and other domains such as setting, flexibility:delivery, and follow-up were not shown to be pragmatic. The flexibility:adherence domain was insufficient to be assessed, and more description is needed in future PATs for this domain as well as the recruitment domain. East Asian countries tended to conduct less pragmatic trials, and there was minimal difference observed across publication years.

### Supplementary Information

**Supplementary Material 1.**

**Supplementary Material 2.**

## Data Availability

All the data analysed/generated in this study is involved in this published article.

## Abbreviations

PAT: Pragmatic acupuncture trials
PRECIS-2: PRagmatic Explanatory Continuum Indicator Summary-2
AMED: Allied and Complementary Medicine Database
RCT: Randomised controlled trial
GP: General practice
ITT: Intention-to-treat
STRICTA: Standards for Reporting Interventions in Clinical Trials of Acupuncture
