# Supplementary material for: PRECIS-2 analysis of pragmatic acupuncture trials: a systematic review
Source: BMC Complement Med Ther. 2024 May 3;24:181. doi: 10.1186/s12906-024-04473-7 (PMC11067076; doi:10.1186/s12906-024-04473-7)
Supplement: Supplementary file 1 — Supplementary Material 1. [file 12906_2024_4473_MOESM1_ESM.pdf]

Table S1. PRISMA 2020 Checklist

Section and Topic	Item #	Checklist item	Location where item is reported
**TITLE**			
Title	1	Identify the report as a systematic review.	1
**ABSTRACT**			
Abstract	2	See the PRISMA 2020 for Abstracts checklist.	2
**INTRODUCTION**			
Rationale	3	Describe the rationale for the review in the context of existing knowledge.	3
Objectives	4	Provide an explicit statement of the objective(s) or question(s) the review addresses.	3
**METHODS**			
Eligibility criteria	5	Specify the inclusion and exclusion criteria for the review and how studies were grouped for the syntheses.	4
Information sources	6	Specify all databases, registers, websites, organisations, reference lists and other sources searched or consulted to identify studies. Specify the date when each source was last searched or consulted.	3-4
Search strategy	7	Present the full search strategies for all databases, registers and websites, including any filters and limits used.	3-4
Selection process	8	Specify the methods used to decide whether a study met the inclusion criteria of the review, including how many reviewers screened each record and each report retrieved, whether they worked independently, and if applicable, details of automation tools used in the process.	4
Data collection process	9	Specify the methods used to collect data from reports, including how many reviewers collected data from each report, whether they worked independently, any processes for obtaining or confirming data from study investigators, and if applicable, details of automation tools used in the process.	4-5
Data items	10a	List and define all outcomes for which data were sought. Specify whether all results that were compatible with each outcome domain in each study were sought (e.g. for all measures, time points, analyses), and if not, the methods used to decide which results to collect.	N/A
	10b	List and define all other variables for which data were sought (e.g. participant and intervention characteristics, funding sources). Describe any assumptions made about any missing or unclear information.	N/A
Study risk of bias assessment	11	Specify the methods used to assess risk of bias in the included studies, including details of the tool(s) used, how many reviewers assessed each study and whether they worked independently, and if applicable, details of automation tools used in the process.	N/A
Effect measures	12	Specify for each outcome the effect measure(s) (e.g. risk ratio, mean difference) used in the synthesis or presentation of results.	N/A
Synthesis methods	13a	Describe the processes used to decide which studies were eligible for each synthesis (e.g. tabulating the study intervention characteristics and comparing against the planned groups for each synthesis (item #5)).	N/A
	13b	Describe any methods required to prepare the data for presentation or synthesis, such as handling of missing summary statistics, or data conversions.	N/A
	13c	Describe any methods used to tabulate or visually display results of individual studies and syntheses.	N/A
	13d	Describe any methods used to synthesize results and provide a rationale for the choice(s). If meta-analysis was performed, describe the	N/A

Section and Topic	Item #	Checklist item	Location where item is reported
		model(s), method(s) to identify the presence and extent of statistical heterogeneity, and software package(s) used.	
	13e	Describe any methods used to explore possible causes of heterogeneity among study results (e.g. subgroup analysis, meta-regression).	N/A
	13f	Describe any sensitivity analyses conducted to assess robustness of the synthesized results.	N/A
Reporting bias assessment	14	Describe any methods used to assess risk of bias due to missing results in a synthesis (arising from reporting biases).	N/A
Certainty assessment	15	Describe any methods used to assess certainty (or confidence) in the body of evidence for an outcome.	N/A
**RESULTS**			
Study selection	16a	Describe the results of the search and selection process, from the number of records identified in the search to the number of studies included in the review, ideally using a flow diagram.	5
	16b	Cite studies that might appear to meet the inclusion criteria, but which were excluded, and explain why they were excluded.	N/A
Study characteristics	17	Cite each included study and present its characteristics.	Additional File 1
Risk of bias in studies	18	Present assessments of risk of bias for each included study.	N/A
Results of individual studies	19	For all outcomes, present, for each study: (a) summary statistics for each group (where appropriate) and (b) an effect estimate and its precision (e.g. confidence/credible interval), ideally using structured tables or plots.	N/A
Results of syntheses	20a	For each synthesis, briefly summarise the characteristics and risk of bias among contributing studies.	N/A
	20b	Present results of all statistical syntheses conducted. If meta-analysis was done, present for each the summary estimate and its precision (e.g. confidence/credible interval) and measures of statistical heterogeneity. If comparing groups, describe the direction of the effect.	N/A
	20c	Present results of all investigations of possible causes of heterogeneity among study results.	N/A
	20d	Present results of all sensitivity analyses conducted to assess the robustness of the synthesized results.	N/A
Reporting biases	21	Present assessments of risk of bias due to missing results (arising from reporting biases) for each synthesis assessed.	N/A
Certainty of evidence	22	Present assessments of certainty (or confidence) in the body of evidence for each outcome assessed.	N/A
**DISCUSSION**			
Discussion	23a	Provide a general interpretation of the results in the context of other evidence.	11
	23b	Discuss any limitations of the evidence included in the review.	N/A
	23c	Discuss any limitations of the review processes used.	11
	23d	Discuss implications of the results for practice, policy, and future research.	11
**OTHER INFORMATION**			

Section and Topic	Item #	Checklist item	Location where item is reported
Registration and protocol	24a	Provide registration information for the review, including register name and registration number, or state that the review was not registered.	3
	24b	Indicate where the review protocol can be accessed, or state that a protocol was not prepared.	3
	24c	Describe and explain any amendments to information provided at registration or in the protocol.	3
Support	25	Describe sources of financial or non-financial support for the review, and the role of the funders or sponsors in the review.	14
Competing interests	26	Declare any competing interests of review authors.	13
Availability of data, code and other materials	27	Report which of the following are publicly available and where they can be found: template data collection forms; data extracted from included studies; data used for all analyses; analytic code; any other materials used in the review.	13

Table S2. Characteristics of included studies

First author	Year	Country	Article type	Disease or condition	Experimental intervention	Control intervention	Primary outcome (clinical)
Tsukayama[1]	2002	Japan	Preliminary study	Low back pain	Electroacupuncture	TENS	Pain relief scale (VAS)
Edwards[2]	2003	UK	Original study	Myofascial pain	Group A: superficial dry needling + stretching exercises<br>Group B: stretching exercises	No treatment	Short form McGil Pain Questionnaire, pressure pain threshold
Vickers[3]	2004	USA	Original study	Headache	Acupuncture + usual care	Usual care	Headache score
Kim[4]	2006	Korea	Original study	Osteoarthritis of the knee	Individualised acupuncture	Standardised acupuncture	Western Ontario and McMaster Universities Osteoarthritis Index
Price[5]	2006	UK	Protocol for pilot study	Breast cancer	Acupuncture + usual care	Usual care	Brief Fatigue Inventory
Thomas[6]	2006	UK	Original study	Non-specific low back pain	Acupuncture	Usual care	SF-36 bodily pain
Witt[7]	2006	Germany	Original study	Chronic low back pain	Group A: acupuncture + usual care<br>Group B: (non-randomised) acupuncture + usual care	Usual care	Hannover Functional Ability Questionnaire
Vas[8]	2008	Spain	Protocol	Migraine	Acupuncture + usual care	Group A: sham acupuncture + usual care<br>Group B: usual care	Difference in the number of days with migraine
Kindberg[9]	2008	Denmark	Original study	Pain during postpartum surgical repair	Ear acupuncture + acupuncture	Local anaesthetics	Pain experienced during surgical repair reported by women at 24–48 hours postpartum
Brinkhaus[10]	2008	Germany	Original study	Allergic rhinitis	Group A: acupuncture + usual care<br>Group B (non-randomised): acupuncture + usual care	Usual care	Rhinitis Quality of Life Questionnaire
Reynolds[11]	2008	UK	Pilot study	Irritable bowel syndrome	Acupuncture + usual care	Usual care	IBS symptom severity score
Fleckenstein[12]	2009	Germany	Protocol	Herpes zoster pain	Acupuncture + usual care	Group A: gabapentine + usual care<br>Group B: sham laser acupuncture + usual care	VAS

Schroer[13]	2009	UK	Protocol for pilot study	Depression	Acupuncture + usual care	Group A: usual care<br>Group B: usual care + non-directive counselling	Beck Depression Inventory
Lansdown[14]	2009	UK	Pilot study	Pain and osteoarthritis of the knee	Acupuncture + usual care	Usual care	Western Ontario and McMaster University Osteoarthritis Index
Borud[15]	2009	Norway	Original study	Hot flushes among menopausal women	Acupuncture + one-page information leaflet	One-page information leaflet	Mean hot flash frequency
Sundberg[16]	2009	Sweden	Pilot study	Back and neck pain	Integrative management (Swedish massage, manipulative therapy, Shiatsu, acupuncture, qigong, maximum 3 combination) + usual care	Usual care	SF-36
Kim[17]	2010	Korea	Original study	Hot flashes in perimenopausal and postmenopausal women	Acupuncture + usual care	Usual care	Mean reduction in average 24-hour hot flash score
Molsberger[18]	2010	Germany	Original study	Chronic shoulder pain	Acupuncture	Group A: sham acupuncture<br>Group B: conventional orthopaedic therapy	VAS
Liodden[19]	2011	Norway	Original study	Postoperative vomiting following paediatric tonsillectomy or adenoidectomy	Acupuncture + acupressure	Usual care	Occurrence of vomiting or retching
Huang[20]	2011	UK	Pilot study	Chronic stress	Acupuncture	Group A: general consultation<br>Group B: waiting list	PSS-14, the Measure Yourself Medical Outcome Profile
Hinman[21]	2012	Australia	Protocol	Chronic knee pain	Group A: acupuncture<br>Group B: laser acupuncture	Group A: sham laser acupuncture<br>Group B: waiting list	Pain NRS, Western Ontario and McMaster University Osteoarthritis Index
Molassiotis[22]	2012	UK	Original study	Cancer-related fatigue in patients with breast cancer	Acupuncture + usual care	Usual care	Multidimensional Fatigue Inventory
Macpherson[23]	2012	UK	Original study	Irritable bowel syndrome	Acupuncture + usual care	Usual care	IBS Symptom Severity Score

Painovich[24]	2012	USA	Original study	All hospitalised patients	Acupuncture + usual care	Usual care	Length of stay
Li[25]	2012	China	Protocol	Functional constipation	Group A: acupuncture (Back-Shu and Front-Mu acupoints)<br>Group B: acupuncture (He-Sea and Lower He-Sea acupoints)<br>Group C: acupuncture (Combining used Back-Shu, Front-Mu, He-Sea, and Lower He-Sea acupoints)	Mosapride Citrate	Number of times of defecation
Duncan[26]	2012	China	Original study	Children with cerebral palsy	Acupuncture + usual care	Usual care	Gross Motor Function Measure, Pediatric Evaluation of Disability Inventory
Chen[27]	2013	China	Protocol	Migraine without aura	Individualised acupuncture	Group A: sham acupuncture<br>Group B: waiting list	Frequency of migraine attacks
Kim[28]	2013	Korea	Protocol	Knee osteoarthritis	Group A: individualised acupuncture<br>Group B: standard acupuncture	Group A: sham acupuncture<br>Group B: waiting list	Western Ontario and McMaster University Osteoarthritis Index
Arvidsdotter [29]	2013	Sweden	Original study	Psychological distress	Group A: acupuncture<br>Group B: acupuncture + salutogenic dialogue	Usual care	Hospital Anxiety and Depression Scale
Molassiotis[30]	2013	UK	Original study	Cancer-related fatigue	Group A: acupuncturist-delivered acupuncture<br>Group B: self-delivered acupuncture	No treatment	Multidimensional Fatigue Inventory
Dascanio[31]	2014	UK	Feasibility test, randomised cohort study	Low back pain	Group A: acupuncture + usual care<br>Group B: manual therapy + usual care<br>Group C: acupuncture + manual therapy + usual care	Usual care	Roland Morris Disability Questionnaire
Wang[32]	2014	China	Original study	Depression in hospital	Acupuncture + SSRI	SSRI	Hamilton Depression Rating Scale-17
Gao[33]	2015	China	Protocol	Simple obesity and overweight	Electro-acupuncture + lifestyle modification	Lifestyle modification	Body Mass Index
Deng[34]	2015	China	Protocol	Posterior circulation	Group A: acupuncture twirling	N/A	Vertebrobasilar System

				ischemia with vertigo	at a frequency of 60 times per minute toward the contralateral outer canthus at a depth of 0.5 to 0.8 cun.<br>Group B: acupuncture twirling at a frequency of 60 times per minute toward the Adam's apple at a depth of 0.5 to 0.8 cun.<br>Group C: acupuncture twirling at a frequency of 120 times per minute toward the contralateral outer canthus at a depth of 0.5 to 0.8 cun.<br>Group D: acupuncture twirling at a frequency of120 times per minute toward the Adam's apple at a depth of 0.5 to 0.8 cun		Ischemic Neurological Impairment Scale, UCLA Dizziness Questionnaire, Activities of Daily Living Scale, Psychological and Social Adaptation Scale, transcranial doppler, carotid ultrasonography, changes in cerebral oxygenation
Macpherson[35]	2015	UK	Original study	Chronic neck pain	Group A: acupuncture + usual care<br>Group B: Alexander lessons + usual care	Usual care	Northwick Park Neck Pain Questionnaire
Bosch[36]	2015	Netherlands	Original study	Mood and working memory in patients with depression and schizophrenia	Acupuncture	Normal psychiatric treatment	Beck Depression Inventory-II, working memory task
Moss[37]	2015	USA	Original study	Acute sore throat	Ear acupuncture + usual care	Usual care	Pain VAS
Shi[38]	2015	China	Original study	Vascular dementia	Group A: acupuncture + usual care<br>Group B (non-randomised): acupuncture + usual care	Usual care	Mini-mental State Examination, Activity of Daily Living Scales, Dementia Quality of Life Questionnaire
Liodden[39]	2015	Norway	Original study	Postoperative vomiting following paediatric tonsillectomy or adenoidectomy	Acupuncture + usual care	Usual care	Nausea and vomiting during 24 h postoperatively
Mafetoni[40]	2015	Brazil	Original study	Labour and caesarean section	Acupressure	Group A: touch<br>Group B: usual care	Duration and induction of labour

				rate			
Shuji[41]	2015	Japan	Original study	Various symptoms from haemodialysis patient	Acupuncture (M-test)	Waiting list	VAS for each symptom, EQ-5D
Liu[42]	2015	New Zealand	Protocol for pilot study	Chronic low back pain	Group A: acupuncture (4 sessions) + usual care<br>Group B: acupuncture (7 sessions) + usual care<br>Group C: acupuncture (10 sessions) + usual care	N/A	Roland-Morris Disability Questionnaire, VAS, SF-12V2, three separate questions taken from the United States National Health Interview Survey, Patient satisfaction question
Witt[43]	2015	Italy	Original study	Breast Cancer	Complementary medicine	Usual care	Functional Assessment of Cancer Therapy-Breast
Mafetoni[44]	2016	Brazil	Original study	Labour pain	Ear acupuncture + usual care	Group A: placebo acupuncture + usual care<br>Group B: usual care	Pain VAS
Li[45]	2016	China	Original study	Severe acute pancreatitis accompanied with paralytic ileus	Electroacupuncture + usual care	Usual care	VAS-abdominal distension and pain severity scale
Trevelyan[46]	2016	UK	Feasibility test	Phantom limb syndrome	Acupuncture or electroacupuncture + usual care	Usual care	Pain NRS
Betts[47]	2016	Australia	Feasibility test	Threatened miscarriage	Acupuncture + self-care advice	Touch to non-acupuncture point + self-care advice	Ongoing pregnancy rate
Conboy[48]	2016	USA	Original study	Gulf war illness	Acupuncture	Usual care	SF-36
Avis[49]	2016	USA	Original study	Menopause	Acupuncture	Usual care	Change in frequency and severity of hot flashes and night sweats
Lesi[50]	2016	Italy	Original study	Hot flashes in women with breast cancer	Acupuncture + enhanced self-care	Enhanced self-care	Hot flash score
Usichenko[51]	2016	Germany	Original study (cross-over design)	Pain and autonomic distress during injection of local anaesthetic	Acupuncture + usual care	Usual care	Verbal rating scale or Wong-Baker Faces Pain Scale
Mafetoni[52]	2016	Brazil	Original study	Labour pain	Acupressure	Group A: touch<br>Group B: waitlist	Pain VAS

Chao[53]	2016	USA	Protocol for pilot study	Pain and symptom management	Acupuncture	Usual care	NRS
Du[54]	2017	China	Protocol	Blood pressure for secondary prevention of stroke	Acupuncture (HuoXueSanFeng acupuncture) + usual care (including XingNaoKaiQiao needling)	Usual care (including XingNaoKaiQiao needling)	Recurrence of stroke
Georgoudis[55]	2017	Greece	Original study	Tension-type headache	Acupuncture + stretching exercise + physiotherapy	Acupuncture + stretching exercise	Mechanical pressure pain threshold
Cohen[56]	2017	Australia	Original study	Analgesia in the emergency department	Group A: acupuncture<br>Group B: acupuncture + usual care	Usual care	Verbal NRS
Brinkhaus[57]	2017	Germany	Original study	Allergic asthma	Group A: acupuncture + usual care<br>Group B (non-randomised): acupuncture + usual care	Usual care	Asthma quality of life questionnaire
Fan[58]	2018	USA	Protocol	Vulvodynia	Group A: acupuncture (pudendal nerve distribution area) + usual care<br>Group B: acupuncture (common meridian or distal points) + usual care	Usual care	Objective pain intensity using the cotton swab test
Heo[59]	2018	Korea	Pilot study	Non-acute low back pain after back surgery	Electroacupuncture + usual care	Usual care	Pain VAS
Qi[60]	2018	China	Original study	Diabetes patients with mild lower-extremity arterial disease	Acupuncture + low-radon hot spring thermal hydrotherapy + usual care + usual care	Usual care	Symptomatic LEAD assessment through ABPI, Edinburgh claudication questionnaire evaluation, leg vascular conductance values, laboratory physical status through lower-body flexibility and aerobic walking endurance measures, self-report measures through the walking impairment questionnaire, EQ-5D,

							Depression Anxiety and Stress Scale 21, SF-12
Bellmann-Strobl[61]	2018	Germany	Protocol	Multiple sclerosis associated fatigue	Group A: acupuncture + usual care<br>Group B: mindfulness-based stress reduction (MBSR) + usual care	Usual care	Fatigue Severity Scale
Blödt[62]	2018	Germany	Original study	Menstrual pain	App-based self-acupressure + usual care	Usual care	Pain NRS
Lin[63]	2019	China	Protocol for pilot study	Osteoarthritis of the knee	Acupuncture (3 sessions per week)	Acupuncture (once per week)	Response rate using NRS and Western Ontario and McMaster University Osteoarthritis Index
Garland[64]	2019	USA	Original study	Insomnia in cancer survivors	Acupuncture	Cognitive behavioural therapy	Insomnia Severity Index
Kwan[65]	2019	Singapor e	Protocol	Axial spondylarthritis	TCM-collaborative model of care + usual care	Usual care	Pain NRS
Chung[66]	2019	Honkong	Original study	Refractory functional dyspepsia	Electroacupuncture + on-demand gastrocaine	On-demand gastrocaine	Binary assessment of adequate relief
Brinkhaus[6 7]	2019	Germany	Original study	Breast cancer	Acupuncture + usual care	Usual care	Quality of life (Functional Assessment of Cancer Therapy-Breast score)
Jang[68]	2019	Korea	Original study	Smoking cessation	Traditional and complementary intervention (including acupuncture) + nicotine replacement therapy + counselling	Nicotine replacement therapy + counselling	Continuous abstinence rate
Zhao[69]	2019	China	Original study	Depression	Group A: Acupuncture + SSRI<br>Group B: Electroacupuncture + SSRI	SSRI	Response rate of HAMD-17 (Hamilton Depression Scale)
Nicolian[70]	2019	France	Original study	Pelvic and low back pain in pregnancy	Acupuncture + usual care	Usual care	Proportion of days with self-assessed pain by NRS ≤ 4/10
Lund[71]	2019	Denmark	Original study	Menopausal symptoms	Western medical acupuncture	Waiting list	Hot flushes scale of MenoScores Questionnaire
Sun[72]	2019	China	Original study	Nail biting	Auricular acupressure + habit reversal treatment	Placebo auricular acupressure + habit reversal treatment	Screen for Child Anxiety Related Emotional Disorders
Bosch[73]	2019	Germany	Original study	Depression	Acupuncture	Waiting list	Beck Depression Inventory-

							II
Noll[74]	2019	USA	Original study	Postoperative patient	Acupressure + usual care	Group A: sham acupressure + usual care<br>Group B: usual care	Quality of recovery-15 questionnaire
Shogo[75]	2019	Japan	Original study	Presenteeism	Acupuncture + usual care	Usual care	WHO-HPQ
Kou[76]	2020	China	Protocol	Acne vulgaris	Acupuncture + topical AA cream	Topical AA cream	Skin lesions score scale
Li[77]	2020	China	Protocol	Erectile dysfunction in post-stroke patients	Acupuncture + rehabilitation training + usual care	Rehabilitation training + usual care	International Erectile Function Index-5
Lei[78]	2020	China	Protocol	Pain caused by prostate cancer	Acupuncture + usual care	Placebo + usual care	Pain NRS
Wang[79]	2020	China	Protocol	Chronic prostatitis/chronic pelvic pain syndrome	Acupuncture combined with tamsulosin hydrochloride sustained-release capsules	Tamsulosin hydrochloride sustained-release capsules	National Institutes of Health's Symptom Score Index score for CP/CPPS
Susana[80]	2020	Spain	Original study	Benign-origin cervical pain	Self-applied acupressure + usual care	Usual care	Pain VAS
Ormsby[81]	2020	Australia	Original study	Antenatal depression	Acupuncture with auricular acupuncture + usual care	Group A: progressive muscle relaxation + usual care<br>Group B: usual care	Edinburgh Postnatal Depression Scale
Lee[82]	2020	Korea	Protocol	Children with cerebral palsy	Integrative medicine rehabilitation (acupuncture, herbal medicine) + usual rehabilitation	Usual rehabilitation	Gross Motor Function Measure-88 score
Zhao[83]	2020	Australia	Protocol (cross-over design)	Cancer-related pain	Acupuncture (body, auricular) + usual care	Usual care	Pain NRS
Reed[84]	2020	Canada	Original study	Cancer pain	Group acupuncture	Individual acupuncture	Brief Pain Inventory
Cai[85]	2021	China	Pilot study	Post-stroke spasticity	Electroacupuncture + usual care	Usual care	Modified Ashworth Scale
Hughes[86]	2021	UK	Feasibility test	Cancer patients undergoing radiotherapy treatment	Acupuncture + usual care	Usual care	Multidimensional Fatigue Inventory
Park[87]	2021	Korea	Protocol	Knee replacement	Acupuncture + usual care	Usual care	Korean version of Western Ontario and McMaster University Osteoarthritis Index
Heo[88]	2021	Korea	Original study	Non-acute pain after	Electroacupuncture + usual care	Usual care	Pain VAS

				back surgery			
Levy[89]	2021	Israel	Original study	Delirium in Older Adults	Acupuncture + usual care	Usual care	Resolution of delirium (time-to-first remission of delirium)
Chen[90]	2021	China	Protocol	Advanced cancer	Acupuncture + usual care	Group A: sham acupuncture + usual care<br>Group B: usual care	Edmonton Symptom Assessment System
Han[91]	2021	Korea	Protocol for pilot study	Lumbar spinal stenosis	Acupotomy + acupuncture + ICT	Acupuncture + ICT	VAS
Ren[92]	2022	China	Protocol	Protracted amphetamine abstinence syndrome	Electroacupuncture	Waitlist	Amphetamine Cessation Symptom Assessment
He[93]	2022	China	Pilot study	Cancer pain	Acupuncture + opioids	Opioids	NRS

Table S3. The scores of PRECIS-2 and control domain of the included studies

First author Year	Eligibility	Recruitment	Setting	Organisation	Flexibility: delivery	Flexibility: adherence	Follow-up	Primary outcome	Primary analysis	Overall	Control
Tsukayama[1] 2002	3	2	3	-	3	-	1	5	1	2.57	3
Edwards[2] 2003	4	5	3	2	Group A: 5<br>Group B: 3	-	4	-	5	4.00	-
Vickers[3] 2004	4	5	5	4	5	-	3	2	5	4.13	-
Kim[4] 2006	1	2	1	3	2	-	2	4	1	2.00	3
Price[5] 2006	-	5	-	5	-	-	1	4	-	3.75	-
Thomas[6] 2006	5	5	5	5	5	-	3	5	1	4.25	5
Witt[7] 2006	3	5	-	5	Group A: 5<br>Group B: 5	-	4	2	5	4.14	5
Vas[8] 2008	1	-	5	5	4	-	1	2	5	3.29	Group A: 1<br>Group B: 5
Kindberg[9] 2008	4	5	3	2	1	-	3	5	5	3.50	4
Brinkhaus[10] 2008	4	5	-	5	Group A: 5<br>Group B: 5	-	4	2	5	4.29	5
Reynolds[11] 2008	4	5	5	5	5	-	3	3	5	4.38	5
Fleckenstein[12] 2009	3	-	-	5	3	-	1	5	-	3.40	Group A: 3<br>Group B: 2
Schroer[13] 2009	3	5	-	5	-	-	1	5	5	4.00	Group A: -<br>Group B: -
Lansdown[14] 2009	4	5	4	5	4.5	-	2	4	5	4.19	5
Borud[15] 2009	1	2	5	5	5	-	2	2	5	3.38	4
Sundberg[16] 2009	5	5	5	2	5	-	4	4	1	3.88	5

Kim[17]<br>2010	2	2	3	5	1	-	4	2	4	2.88	3
Molsberger[18]<br>2010	4	5	5	5	2	-	4	5	4	4.25	Group A: 1<br>Group B: 4
Liodden[19]<br>2011	4	4	3	2	1	-	5	5	5	3.63	3
Huang[20]<br>2011	4	2	-	-	2	-	5	4	5	3.67	Group A: 3<br>Group B: -
Hinman[21]<br>2012	2	1	5	3	Group A: 4<br>Group B: 4	Group A: 2<br>Group B: 2	2	4	5	3.11	Group A: 1<br>Group B: -
Molassiotis[22]<br>2012	3	2	5	5	2	-	5	4	5	3.88	4
Macpherson[23]<br>2012	4	5	5	5	5	-	3	3	5	4.38	5
Painovich[24]<br>2012	5	-	3	4	5	-	5	5	1	4.00	-
Li[25]<br>2012	2	1	3	2	Group A: 1<br>Group B: 1<br>Group C: 1	NR	3	4	4	2.5	3
Duncan[26]<br>2012	5	NR	3	NR	NR	NR	3	2	1	2.8	4
Chen[27]<br>2013	2	-	-	-	2	2	1	2	5	2.50	Group A: 1<br>Group B: 4
Kim[28]<br>2013	3	2	3	3	Group A: 2<br>Group B: 1	-	3	4	5	3.13	Group A: 1<br>Group B: -
Arvidsdotter[29]<br>2013	4	5	5	2	Group A: 1<br>Group B: 2	-	4	3	5	3.63	5
Molassiotis[30]<br>2013	3	5	NR	1	Group A: 2<br>Group B: 1	NR	4	4	1	2.86	4
Dascanio[31]<br>2014	3	5	4	3	Group A: -<br>Group B: 3	-	2	3	5	3.57	-

					Group C: -						
Wang[32] 2014	4	-	1	2	3	-	5	4	1	2.86	3
Gao[33] 2015	2	3	3	1	3	-	2	5	5	3.00	3
Deng[34] 2015	4	2	1	2	Group A: 2<br>Group B: 2<br>Group C: 2<br>Group D: 2	-	3	1	-	2.14	-
Macpherson[35] 2015	3	5	5	5	Group A: 5<br>Group B: 5	-	2	4	5	4.25	5
Bosch[36] 2015	4	-	-	5	5	-	5	-	1	4.00	-
Moss[37] 2015	2	5	1	5	4	-	3	5	5	3.75	3
Shi[38] 2015	3	-	-	5	Group A: 4<br>Group B: 4	-	4	-	1	3.40	4
Liodden[39] 2015	5	-	5	2	1	-	5	5	5	4.00	1
Mafetoni[40] 2015	5	-	3	2	1	-	5	5	5	3.71	Group A: 1<br>Group B: 3
Shuji[41] 2015	-	-	-	2	1	-	3	-	1	1.75	3
Liu[42] 2015	3	1	1	3	2	2	3	3	5	2.56	NA
Witt[43] 2015	5	NR	4	NR	5	NR	4	4	5	4.5	5
Mafetoni[44] 2016	5	5	3	-	1	-	4	5	5	4.00	Group A: 1<br>Group B: -
Li[45] 2016	3	-	3	5	1	2	5	5	1	3.13	4
Trevelyan[46] 2016	4	-	3	2	4	-	2	5	1	3.00	5
Betts[47] 2016	5	4	3	2	4	-	4	5	1	3.50	2

Conboy[48] 2016	4	2	5	3	5	-	3	2.5	1	3.19	5
Avis[49] 2016	3	2	-	4	5	1	1	2	1	2.38	5
Lesi[50] 2016	2.5	4	5	3	3	-	4	2	5	3.56	3
Usichenko[51] 2016	4	-	-	2	1	-	5	5	-	3.40	3
Mafetoni[52] 2016	5	-	3	2	1	-	5	5	5	3.71	Group A: 1<br>Group B: 3
Chao[53] 2016	5	5	3	1	5	NR	3	5	5	4	5
Du[54] 2017	3	2	3	3	1	2	1	5	5	2.78	3
Georgoudis[55] 2017	4	-	-	2.5	1	-	5	2	5	3.25	3
Cohen[56] 2017	4	5	5	5	Group A: 4<br>Group B: 4	-	3	5	1	4.00	5
Brinkhaus[57] 2017	5	-	-	5	Group A: 5<br>Group B: 5	-	4	2.5	1	3.75	5
Fan[58] 2018	3	-	3	5	Group A: 2<br>Group B: 2	-	4	5	-	3.67	5
Heo[59] 2018	2	2	1	3	3	-	4	5	5	3.13	4
Qi[60] 2018	1	-	1	2	2	-	2	1	1	1.43	4
Strobl[61] 2018	2	3	3	1	Group A: 1<br>Group B: 1	-	1	4	4	2.38	5
Blodt[62] 2018	3.5	1	5	5	1	-	4	5	4	3.56	5
Lin[63] 2019	3	2.5	1	4	3	-	3	4	4	3.06	3
Garland[64] 2019	4	4	4	4	3	-	1	4	5	3.63	3
Kwan[65]	2	3	-	5	2	-	1	5	5	3.29	5

2019											
Chung[66] 2019	1.5	2	1	5	1	-	3	5	5	2.94	3
Brinkhaus[67] 2019	4	-	3	1	4	-	3	4	1	2.86	-
Jang[68] 2019	4	2	1	2	1	-	1	2	5	2.25	3
Zhao[69] 2019	3	-	3	5	Group A: 2<br>Group B: 2	-	4	4	5	3.71	4
Nicolian[70] 2019	4	4	3	3	3	-	4	2	5	3.50	5
Lund[71] 2019	2	3	5	5	1	-	3	4	5	3.50	-
Sun[72] 2019	2	1	1	4	1	-	4	4	1	2.25	1
Bosch[73] 2019	4	4	-	5	4	-	4	5	-	4.33	-
Noll[74] 2019	4	5	3	2	1	-	4	4	4	3.38	Group A: 1<br>Group B: -
Shogo[75] 2019	5	2	NR	NR	NR	2	4	4	5	3.67	5
Kou[76] 2020	3	-	1	-	3	-	4	2	-	2.60	3
Li[77] 2020	4	-	1	-	1	-	4	4	-	2.80	5
Lei[78] 2020	4	-	-	-	4	-	4	5	4	4.20	4
Wang[79] 2020	4	-	-	-	1	-	4	4	4	3.40	3
Susana[80] 2020	2	5	5	3	3	-	2	5	1	3.25	-
Ormsby[81] 2020	4	4	4	2	3	4	3	4	5	3.67	Group A: 3<br>Group B: 5
Lee[82] 2020	4	2	-	5	4	2	2	1	5	3.13	4
Zhao[83] 2020	4	3	3	5	2	4	4	5	5	3.89	-

Reed[84] 2020	4	4	3	3	4	2	NR	4	5	3.63	4
Cai[85] 2021	4	-	3	5	3	-	4	2	4	3.57	4
Hughes[86] 2021	3	5	3	2	4	3	3	4	1	3.11	5
Park[87] 2021	4	2	3	5	1	2	2	4	4	3.00	4
Heo[88] 2021	3	2	3	5	3	-	3	5	5	3.63	3
Levy[89] 2021	4	5	3	5	4	-	5	2	5	4.13	4
Chen[90] 2021	4	NR	NR	5	4	NR	2	4	5	4	Group A: 2<br>Group B: 5
Han[91] 2021	3	2	NR	NR	2	NR	3	5	5	3.33	3
Ren[92] 2022	5	NR	4	5	2	2	2	4	5	3.63	NR
He[93] 2022	5	5	3	5	2	NR	4	5	4	4.125	3

Reference of included studies

1. Tsukayama H, Yamashita H, Amagai H, Tanno Y. Randomised controlled trial comparing the effectiveness of electroacupuncture and TENS for low back pain: a preliminary study for a pragmatic trial. Acupunct Med. 2002;20:175–80.

2. Edwards J, Knowles N. Superficial dry needling and active stretching in the treatment of myofascial pain--a randomised controlled trial. Acupunct Med. 2003;21:80–6.

3. Vickers AJ, Rees RW, Zollman CE, McCarney R, Smith CM, Ellis N, et al. Acupuncture for chronic headache in primary care: large, pragmatic, randomised trial. BMJ. 2004;328:744.

4. Kim S-C, Lim J-A, Lee J-D, Lee S-K, Lee S-Y, Moon H-C, et al. A Pilot Study of Acupuncture Treatment for the Osteoarthritis of the Knee Joint on the EBM(Evidence Basement Medicine). Journal of Acupuncture Research. 2006;23:187–215.

5. Price S, Lewith G, Thomas K. Acupuncture care for breast cancer patients during chemotherapy: a feasibility study. Integr Cancer Ther. 2006;5:308–14.

6. Thomas KJ, MacPherson H, Thorpe L, Brazier J, Fitter M, Campbell MJ, et al. Randomised controlled trial of a short course of traditional acupuncture compared with usual care for persistent non-specific low back pain. BMJ. 2006;333:623.

7. Witt CM, Jena S, Selim D, Brinkhaus B, Reinhold T, Wruck K, et al. Pragmatic randomized trial evaluating the clinical and economic effectiveness of acupuncture for chronic low back pain. Am J Epidemiol. 2006;164:487–96.

8. Vas J, Rebollo A, Perea-Milla E, Méndez C, Font CR, Gómez-Río M, et al. Study protocol for a pragmatic randomised controlled trial in general practice investigating the effectiveness of acupuncture against migraine. BMC Complement Altern Med. 2008;8:12.

9. Kindberg S, Klünder L, Strøm J, Henriksen TB. Ear acupuncture or local anaesthetics as pain relief during postpartum surgical repair: a randomised controlled trial. BJOG. 2009;116:569–76.

10. Brinkhaus B, Witt CM, Jena S, Liecker B, Wegscheider K, Willich SN. Acupuncture in patients with allergic rhinitis: a pragmatic randomized trial. Ann Allergy Asthma Immunol. 2008;101:535–43.

11. Reynolds JA, Bland JM, MacPherson H. Acupuncture for irritable bowel syndrome an exploratory randomised controlled trial. Acupunct Med. 2008;26:8–16.

12. Fleckenstein J, Kramer S, Hoffrogge P, Thoma S, Lang PM, Lehmeyer L, et al. Acupuncture in acute herpes zoster pain therapy (ACUZoster) - design and protocol of a randomised controlled trial. BMC Complement Altern Med. 2009;9:31.

13. Schroer S, Macpherson H. Acupuncture, or non-directive counselling versus usual care for the treatment of depression: a pilot study. Trials. 2009;10:3.

14. Lansdown H, Howard K, Brealey S, MacPherson H. Acupuncture for pain and osteoarthritis of the knee: a pilot study for an open parallel-arm randomised controlled trial. BMC Musculoskelet Disord. 2009;10:130.

15. Borud EK, Alraek T, White A, Fonnebo V, Eggen AE, Hammar M, et al. The Acupuncture on Hot Flushes Among Menopausal Women (ACUFLASH) study, a randomized controlled trial. Menopause. 2009;16:484–93.

16. Sundberg T, Petzold M, Wändell P, Rydén A, Falkenberg T. Exploring integrative medicine for back and neck pain - a pragmatic randomised clinical pilot trial. BMC Complementary and Alternative Medicine. 2009;9:33.

17. Kim KH, Kang KW, Kim DI, Kim HJ, Yoon HM, Lee JM, et al. Effects of acupuncture on hot flashes in perimenopausal and postmenopausal women--a multicenter randomized clinical trial. Menopause. 2010;17:269–80.

18. Molsberger AF, Schneider T, Gotthardt H, Drabik A. German Randomized Acupuncture Trial for chronic shoulder pain (GRASP) - a pragmatic, controlled, patient-blinded, multi-centre trial in an outpatient care environment. Pain. 2010;151:146–54.

19. Liodden I, Howley M, Grimsgaard AS, Fønnebø VM, Borud EK, Alraek T, et al. Perioperative acupuncture and postoperative acupressure can prevent postoperative vomiting following paediatric tonsillectomy or adenoidectomy: a pragmatic randomised controlled trial. Acupunct Med. 2011;29:9–15.

20. Huang W, Howie J, Taylor A, Robinson N. An investigation into the effectiveness of traditional Chinese acupuncture (TCA) for chronic stress in adults: a randomised controlled pilot study. Complement Ther Clin Pract. 2011;17:16–21.

21. Hinman RS, McCrory P, Pirotta M, Relf I, Crossley KM, Reddy P, et al. Efficacy of acupuncture for chronic knee pain: protocol for a randomised controlled trial using a Zelen design. BMC Complement Altern Med. 2012;12:161.

22. Molassiotis A, Bardy J, Finnegan-John J, Mackereth P, Ryder DW, Filshie J, et al. Acupuncture for cancer-related fatigue in patients with breast cancer: a pragmatic randomized controlled trial. J Clin Oncol. 2012;30:4470–6.

23. MacPherson H, Tilbrook H, Bland JM, Bloor K, Brabyn S, Cox H, et al. Acupuncture for irritable bowel syndrome: primary care based pragmatic randomised controlled trial. BMC Gastroenterol. 2012;12:150.

24. Painovich J, Herman PM. Acupuncture in the inpatient acute care setting: a pragmatic, randomized control trial. Evid Based Complement Alternat Med. 2012;2012:309762.

25. Li Y, Zheng H, Zeng F, Zhou S, Zhong F, Zheng H, et al. Use acupuncture to treat functional constipation: study protocol for a randomized controlled trial. Trials. 2012;13:104.

26. Duncan B, Shen K, Zou L-P, Han T-L, Lu Z-L, Zheng H, et al. Evaluating intense rehabilitative therapies with and without acupuncture for children with cerebral palsy: a randomized controlled trial. Arch Phys Med Rehabil. 2012;93:808–15.

27. Chen J, Zhao L, Zheng H, Li Y, Yang M, Chang X, et al. Evaluating the prophylaxis and long-term effectiveness of acupuncture for migraine without aura: study protocol for a randomized controlled trial. Trials. 2013;14:361.

28. Kim E-J, Lim C-Y, Lee E-Y, Lee S-D, Kim K-S. Comparing the effects of individualized, standard, sham and no acupuncture in the treatment of knee osteoarthritis: a multicenter randomized controlled trial. Trials. 2013;14:129.

29. Arvidsdotter T, Marklund B, Taft C. Effects of an integrative treatment, therapeutic acupuncture and conventional treatment in alleviating psychological distress in primary care patients--a pragmatic randomized controlled trial. BMC Complement Altern Med. 2013;13:308.

30. Molassiotis A, Bardy J, Finnegan-John J, Mackereth P, Ryder WD, Filshie J, et al. A randomized, controlled trial of acupuncture self-needling as maintenance therapy for cancer-related fatigue after therapist-delivered acupuncture. Ann Oncol. 2013;24:1645–52.

31. Dascanio V, Birks Y, Clark L, Fairhurst C, MacPherson H, Torgerson DJ. Randomized cohort trial was shown to be feasible for evaluating treatments in low back pain. J Clin Epidemiol. 2014;67:940–6.

32. Wang T, Wang L, Tao W, Chen L. Acupuncture combined with an antidepressant for patients with depression in hospital: a pragmatic randomised controlled trial. Acupunct Med. 2014;32:308–12.

33. Gao Z, Yu Z, Song Z-X, Zhang C-R, Wang Y-S, Wu Y-F, et al. Comparative effectiveness of electro-acupuncture plus lifestyle modification treatment for patients with simple obesity and overweight: study protocol for a randomized controlled trial. Trials. 2015;16:525.

34. Deng S-Z, Zhao X-F, Huang L-H, He S, Wen Y, Zhang C, et al. The quantity-effect relationship and physiological mechanisms of different acupuncture manipulations on posterior circulation ischemia with vertigo: study protocol for a randomized controlled trial. Trials. 2015;16:152.

35. MacPherson H, Tilbrook H, Richmond S, Woodman J, Ballard K, Atkin K, et al. Alexander Technique Lessons or Acupuncture Sessions for Persons With Chronic Neck Pain: A Randomized Trial. Ann Intern Med. 2015;163:653–62.

36. Bosch P, van den Noort M, Yeo S, Lim S, Coenen A, van Luijtelaar G. The effect of acupuncture on mood and working memory in patients with depression and schizophrenia. J Integr Med. 2015;13:380–90.

37. Moss DA, Crawford P. Ear Acupuncture for Acute Sore Throat: A Randomized Controlled Trial. J Am Board Fam Med. 2015;28:697–705.

38. Shi G-X, Li Q-Q, Yang B-F, Liu Y, Guan L-P, Wu M-M, et al. Acupuncture for Vascular Dementia: A Pragmatic Randomized Clinical Trial. ScientificWorldJournal. 2015;2015:161439.

39. Liodden I, Sandvik L, Valeberg BT, Borud E, Norheim AJ. Acupuncture versus usual care for postoperative nausea and vomiting in children after tonsillectomy/adenoidectomy: a pragmatic, multicentre, double-blinded, randomised trial. Acupunct Med. 2015;33:196–203.

40. Mafetoni RR, Shimo AKK. Effects of acupressure on progress of labor and cesarean section rate: randomized clinical trial. Rev Saude Publica. 2015;49:9.

41. Ono S, Mukaino Y. Efficacy and Cost Effectiveness of the Acupuncture Treatment Using a New Skin Stimulus Tool Called M-Test Which Is a Measure Based on Symptoms Accompanied with Body Movements: A Pragmatic RCT Targeting Hemodialysis Patients. Evid Based Complement Alternat Med. 2015;2015:802846.

42. Liu L, Skinner MA, McDonough SM, Taylor KGM, Baxter GD. Does the dose of Traditional Chinese Medicine acupuncture in addition to usual care affect outcomes for adults with chronic low back pain? Protocol for a randomized controlled feasibility study. Physical Therapy Reviews. 2015;20:275–82.

43. Witt CM, Außerer O, Baier S, Heidegger H, Icke K, Mayr O, et al. Effectiveness of an additional individualized multi-component complementary medicine treatment on health-related quality of life in breast cancer patients: a pragmatic randomized trial. Breast Cancer Res Treat. 2015;149:449–60.

44. Mafetoni RR, Shimo AKK. Effects of auriculotherapy on labour pain: a randomized clinical trial. Rev Esc Enferm USP. 2016;50:726–32.

45. Li J, Zhao Y, Wen Q, Xue Q, Lv J, Li N. [Electroacupuncture for severe acute pancreatitis accompanied with paralytic ileus:a randomized controlled trial]. Zhongguo Zhen Jiu. 2016;36:1126–30.

46. Trevelyan EG, Turner WA, Summerfield-Mann L, Robinson N. Acupuncture for the treatment of phantom limb syndrome in lower limb amputees: a randomised controlled feasibility study. Trials. 2016;17:519.

47. Betts D, Smith CA, Dahlen HG. Does acupuncture have a role in the treatment of threatened miscarriage? Findings from a feasibility randomised trial and semi-structured participant interviews. BMC Pregnancy Childbirth. 2016;16:298.

48. Conboy L, Gerke T, Hsu K-Y, St John M, Goldstein M, Schnyer R. The Effectiveness of Individualized Acupuncture Protocols in the Treatment of Gulf War Illness: A Pragmatic Randomized Clinical Trial. PLoS One. 2016;11:e0149161.

49. Avis NE, Coeytaux RR, Isom S, Prevette K, Morgan T. Acupuncture in Menopause (AIM) study: a pragmatic, randomized controlled trial. Menopause. 2016;23:626–37.

50. Lesi G, Razzini G, Musti MA, Stivanello E, Petrucci C, Benedetti B, et al. Acupuncture As an Integrative Approach for the Treatment of Hot Flashes in Women With Breast Cancer: A Prospective Multicenter Randomized Controlled Trial (AcCliMaT). J Clin Oncol. 2016;34:1795–802.

51. Usichenko TI, Wolters P, Anders EF, Splieth C. Acupuncture Reduces Pain and Autonomic Distress During Injection of Local Anesthetic in Children: A Pragmatic Crossover Investigation. Clin J Pain. 2016;32:82–6.

52. Mafetoni RR, Shimo AKK. The effects of acupressure on labor pains during child birth: randomized clinical trial. Rev Lat Am Enfermagem. 2016;24:e2738.

53. Chao MT, Chang A, Reddy S, Harrison JD, Acquah J, Toveg M, et al. Adjunctive acupuncture for pain and symptom management in the inpatient setting: protocol for a pilot hybrid effectiveness-implementation study. J Integr Med. 2016;14:228–38.

54. Du Y-Z, Gao X-X, Wang C-T, Zheng H-Z, Lei Y, Wu M-H, et al. Acupuncture lowering blood pressure for secondary prevention of stroke: a study protocol for a multicenter randomized controlled trial. Trials. 2017;18:428.

55. Georgoudis G, Felah B, Nikolaidis P, Damigos D. The effect of myofascial release and microwave diathermy combined with acupuncture versus acupuncture therapy in tension-type headache patients: A pragmatic randomized controlled trial. Physiother Res Int. 2018;23:e1700.

56. Cohen MM, Smit DV, Andrianopoulos N, Ben-Meir M, Taylor DM, Parker SJ, et al. Acupuncture for analgesia in the emergency department: a multicentre, randomised, equivalence and non-inferiority trial. Med J Aust. 2017;206:494–9.

57. Brinkhaus B, Roll S, Jena S, Icke K, Adam D, Binting S, et al. Acupuncture in Patients with Allergic Asthma: A Randomized Pragmatic Trial. J Altern Complement Med. 2017;23:268–77.

58. Fan AY, Alemi SF, Zhu YH, Rahimi S, Wei H, Tian H, et al. Effectiveness of two different acupuncture strategies in patients with vulvodynia: Study protocol for a pilot pragmatic controlled trial. J Integr Med. 2018;16:384–9.

59. Heo I, Hwang M-S, Hwang E-H, Cho J-H, Ha I-H, Shin K-M, et al. Electroacupuncture as a complement to usual care for patients with non-acute low back pain after back surgery: a pilot randomised controlled trial. BMJ Open. 2018;8:e018464.

60. Qi Z, Pang Y, Lin L, Zhang B, Shao J, Liu X, et al. Acupuncture Combined with Hydrotherapy in Diabetes Patients with Mild Lower-Extremity Arterial Disease: A Prospective, Randomized, Nonblinded Clinical Study. Med Sci Monit. 2018;24:2887–900.

61. Bellmann-Strobl J, Pach D, Chang Y, Pasura L, Liu B, Jäger SF, et al. The effectiveness of acupuncture and mindfulness-based stress reduction (MBSR) for patients with multiple sclerosis associated fatigue – A study protocol and its rationale for a randomized controlled trial. European Journal of Integrative Medicine. 2018;20:6–15.

62. Blödt S, Pach D, Eisenhart-Rothe S von, Lotz F, Roll S, Icke K, et al. Effectiveness of app-based self-acupressure for women with menstrual pain compared to usual care: a randomized pragmatic trial. Am J Obstet Gynecol. 2018;218:227.e1-227.e9.

63. Lin L-L, Tu J-F, Shao J-K, Zou X, Wang T-Q, Wang L-Q, et al. Acupuncture of different treatment frequency in knee osteoarthritis: a protocol for a pilot randomized clinical trial. Trials. 2019;20:423.

64. Garland SN, Xie SX, DuHamel K, Bao T, Li Q, Barg FK, et al. Acupuncture Versus Cognitive Behavioral Therapy for Insomnia in Cancer Survivors: A Randomized Clinical Trial. J Natl Cancer Inst. 2019;111:1323–31.

65. Kwan YH, Fong W, Ang XL, Tan CS, Tai BC, Huang Y, et al. Traditional Chinese medicine (TCM) collaborative care for patients with axial spondyloarthritis (AcuSpA): protocol for a pragmatic randomized controlled trial. Trials. 2019;20:46.

66. Chung VC, Wong CH, Wu IX, Ching JY, Cheung WK, Yip BH, et al. Electroacupuncture plus on-demand gastrocaine for refractory functional dyspepsia: Pragmatic randomized trial. J Gastroenterol Hepatol. 2019;34:2077–85.

67. Brinkhaus B, Kirschbaum B, Stöckigt B, Binting S, Roll S, Carstensen M, et al. Prophylactic acupuncture treatment during chemotherapy with breast cancer: a randomized pragmatic trial with a retrospective nested qualitative study. Breast Cancer Res Treat. 2019;178:617–28.

68. Jang S, Lee JA, Jang B-H, Shin Y-C, Ko S-G, Park S. Clinical Effectiveness of Traditional and Complementary Medicine Interventions in Combination with Nicotine Replacement Therapy on Smoking Cessation: A Randomized Controlled Pilot Trial. J Altern Complement Med. 2019;25:526–34.

69. Zhao B, Li Z, Wang Y, Ma X, Wang X, Wang X, et al. Manual or electroacupuncture as an add-on therapy to SSRIs for depression: A randomized controlled trial. J Psychiatr Res. 2019;114:24–33.

70. Nicolian S, Butel T, Gambotti L, Durand M, Filipovic-Pierucci A, Mallet A, et al. Cost-effectiveness of acupuncture versus standard care for pelvic and low back pain in pregnancy: A randomized controlled trial. PLoS One. 2019;14:e0214195.

71. Lund KS, Siersma V, Brodersen J, Waldorff FB. Efficacy of a standardised acupuncture approach for women with bothersome menopausal symptoms: a pragmatic randomised study in primary care (the ACOM study). BMJ Open. 2019;9:e023637.

72. Sun D, Reziwan K, Wang J, Zhang J, Cao M, Wang X, et al. Auricular Acupressure Improves Habit Reversal Treatment for Nail Biting. J Altern Complement Med. 2019;25:79–85.

73. Bosch P, Lim S, Staudte H, Lee S-H, Yeo S, Litscher D, et al. Gender Differences in the Acupuncture Treatment of Patients with Depression. Dtsch Z Akupunkt. 2019;62:160–5.

74. Noll E, Shodhan S, Romeiser JL, Madariaga MC, Page C, Santangelo D, et al. Efficacy of acupressure on quality of recovery after surgery: Randomised controlled trial. Eur J Anaesthesiol. 2019;36:557–65.

75. MIYAZAKI Shogo, MINAKAWA Yoichi, SAWAZAKI Kenta, IIMURA Kaori, WAKI Hideaki, TAHARA Iori, et al. Can Allowance for Acupuncture Treatment Benefit Office Workers' Presenteeism? Zen Nihon Shinkyu Gakkai zasshi (Journal of the Japan Society of Acupuncture and Moxibustion). 2019;69:254–65.

76. Kou L, Yu N, Ren J, Yang B, Tao Y. Observation for clinical effect of acupuncture combined with conventional therapy in the treatment of acne vulgaris. Medicine (Baltimore). 2020;99:e19764.

77. Li Y, Yu X, Liu R, Wang J, Deng S, Liu B, et al. Acupuncture for erectile dysfunction in post-stroke patients: Study Protocol Clinical Trial (SPIRIT Compliant). Medicine (Baltimore). 2020;99:e19718.

78. Lei Y, Duan Y, Wang J, Yu X, Deng S, Liu R, et al. A randomized controlled trial for acupuncture combined with conventional therapy in the treatment of pain caused by prostate cancer: Study protocol clinical trial (SPIRIT compliant). Medicine (Baltimore). 2020;99:e19609.

79. Wang J-S, Yang J, Deng S, Yu X-D, Bao B-H, Liu R-J, et al. Acupuncture combined with tamsulosin hydrochloride sustained-release capsule in the treatment of chronic prostatitis/chronic pelvic pain syndrome: A study protocol for a randomized controlled trial. Medicine (Baltimore). 2020;99:e19540.

80. Susana CT, Maria TML, Pilar DS, María MMT, Pilar MS, Valentín MG, et al. Effectiveness of self-applied acupressure for cervical pain of benign origin (EDIDO-CUH): a randomized controlled clinical trial. Acupunct Med. 2021;39:441–51.

81. Ormsby SM, Smith CA, Dahlen HG, Hay PJ. The feasibility of acupuncture as an adjunct intervention for antenatal depression: a pragmatic randomised controlled trial. J Affect Disord. 2020;275:82–93.

82. Lee M-J, Yun Y-J, Yu S-A, Shin Y-B, Kim S-Y, Han J-H. Integrative medicine rehabilitation for children with cerebral palsy: a study protocol for a multicenter pragmatic randomized controlled trial. Trials. 2020;21:723.

83. Zhao Q, Zheng S, Delaney GP, Moylan E, Agar MR, Koh E-S, et al. Acupuncture for Cancer Related Pain: Protocol for a Pragmatic Randomised Wait-List Controlled Trial. Integr Cancer Ther. 2020;19:1534735420976579.

84. Reed EN, Landmann J, Oberoi D, Piedalue K-AL, Faris P, Carlson LE. Group versus Individual Acupuncture (AP) for Cancer Pain: A Randomized Noninferiority Trial. Evidence-based Complementary & Alternative Medicine (eCAM). 2020;:1–12.

85. Cai Y, Zhang CS, Zhang AL, Da Costa C, Xue CC, Wen Z. Electroacupuncture for Poststroke Spasticity: Results of a Pilot Pragmatic Randomized Controlled Trial. J Pain Symptom Manage. 2021;61:305–14.

86. Hughes J, Gyertson K, Carballo L, Plant H, Sharman M, Ruane K, et al. A feasibility trial of acupuncture in cancer patients undergoing radiotherapy treatment. Complement Ther Clin Pract. 2021;43:101372.

87. Park T-Y, Kim H-J, Lee J-H, Sunwoo Y-Y, Do K-S, Han S-N, et al. Efficacy and safety of acupuncture treatment as an adjunctive therapy after knee replacement: Single-center, pragmatic, randomized, assessor blinded, pilot study. Medicine (Baltimore). 2021;100:e24941.

88. Heo I, Shin B-C, Cho J-H, Ha I-H, Hwang E-H, Lee J-H, et al. Multicentre randomised controlled clinical trial of electroacupuncture with usual care for patients with non-acute pain after back surgery. Br J Anaesth. 2021;126:692–9.

89. Levy I, Gavrieli S, Hefer T, Attias S, Schiff A, Oliven R, et al. Acupuncture Treatment of Delirium in Older Adults Hospitalized in Internal Medicine Departments: An Open-Label Pragmatic Randomized-Controlled Trial. J Geriatr Psychiatry Neurol. 2021;:891988721996804.

90. Chen H, So TH, Cho WC-S, Qin Z, Ma CH, Li SG, et al. The Adjunctive Effect of Acupuncture for Advanced Cancer Patients in a Collaborative Model of Palliative Care: Study Protocol for a 3-Arm Randomized Trial. Integr Cancer Ther. 2021;20:15347354211012748.

91. Han JH, Lee H-J, Woo SH, Park Y-K, Choi G-Y, Heo ES, et al. Effectiveness and safety of acupotomy on lumbar spinal stenosis: A pragmatic randomized, controlled, pilot clinical trial: A study protocol. Medicine (Baltimore). 2021;100:e28175.

92. Ren H, Zeng Y, Zhang M, Zhang S, Chen Z, Wu B, et al. Electro-acupuncture for protracted amphetamine abstinence syndrome: study protocol for a pragmatic randomized controlled trial. Trials. 2022;23:216.

93. He Y, Zhang H, Li Y, Long S, Xiao S, May BH, et al. Acupuncture combined with opioids for cancer pain: a pilot pragmatic randomized controlled trial. Acupuncture in Medicine. 2022;40:133–41.
