# Supplementary material for: PRECIS-2 analysis of pragmatic acupuncture trials: a systematic review
Source: BMC Complement Med Ther. 2024 May 3;24:181. doi: 10.1186/s12906-024-04473-7 (PMC11067076; doi:10.1186/s12906-024-04473-7)
Supplement: Supplementary file 2 — Supplementary Material 2. [file 12906_2024_4473_MOESM2_ESM.pdf]

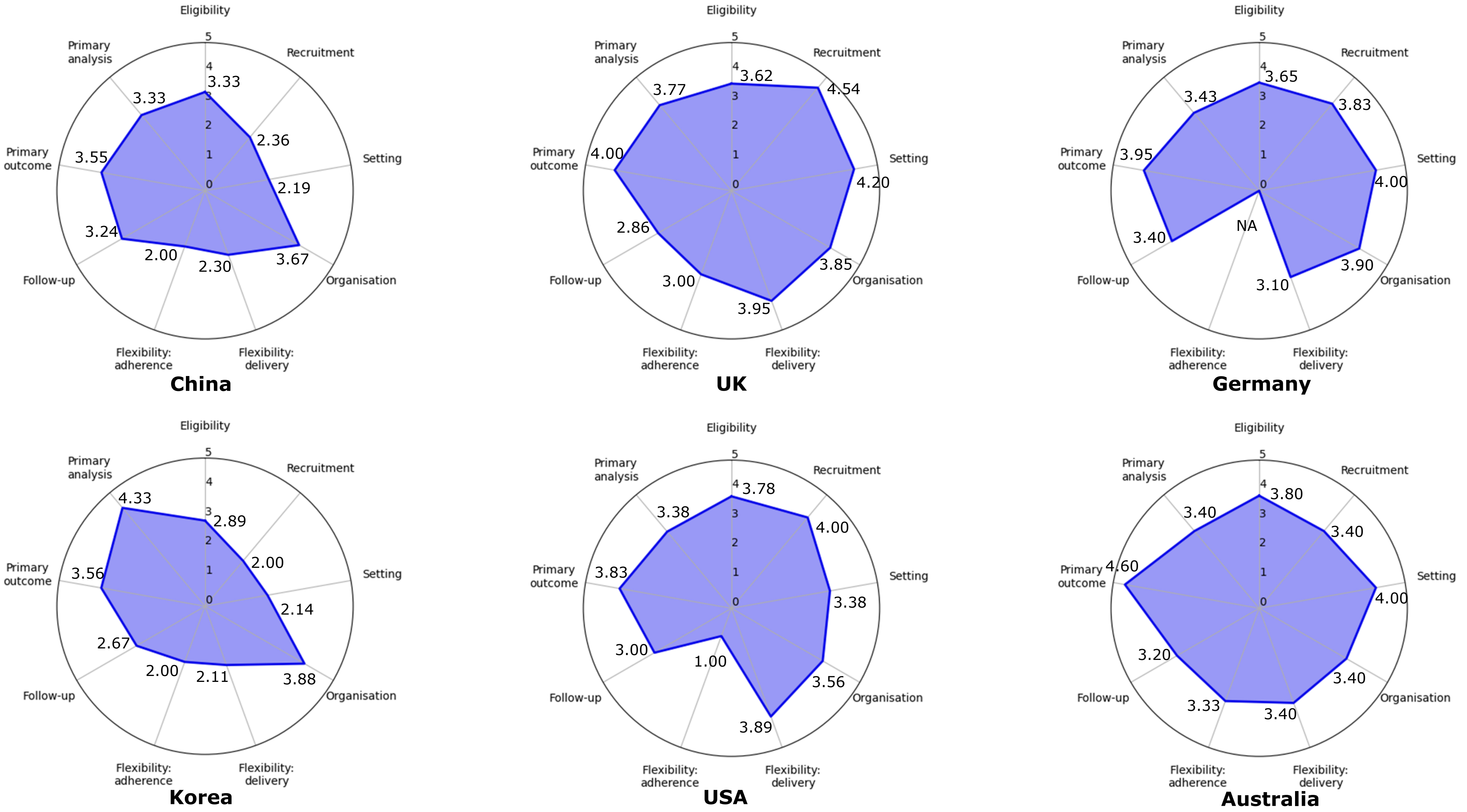

Figure S1. PRECIS-2 score by countries with above 5 studies published

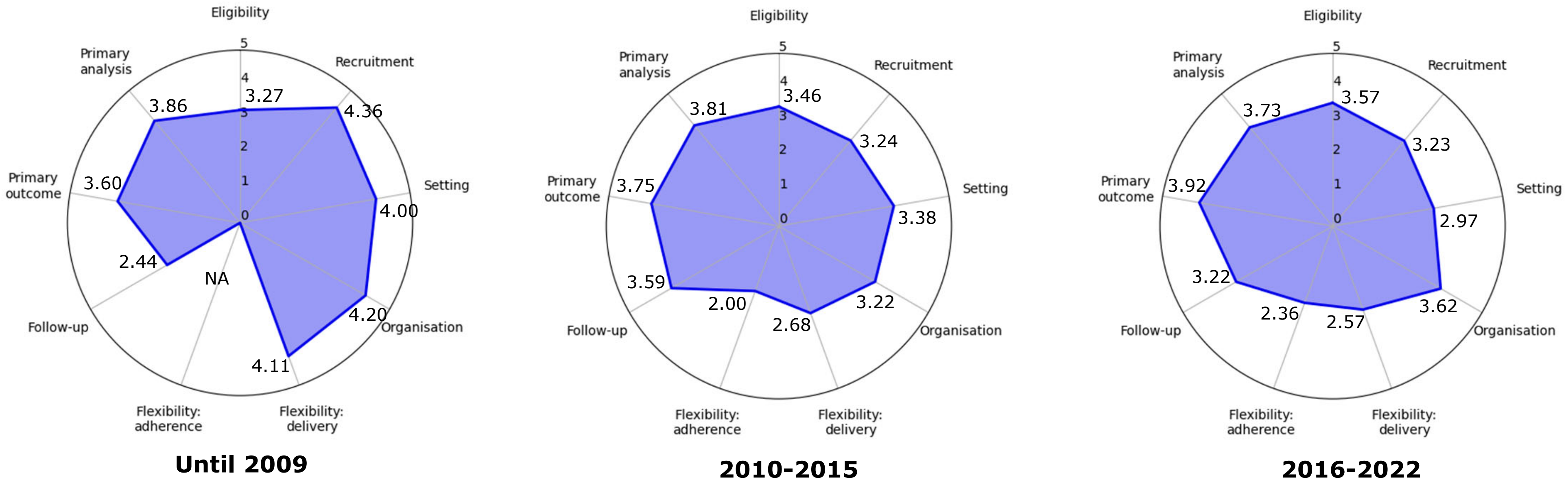

Figure S2. PRECIS-2 score by publication year

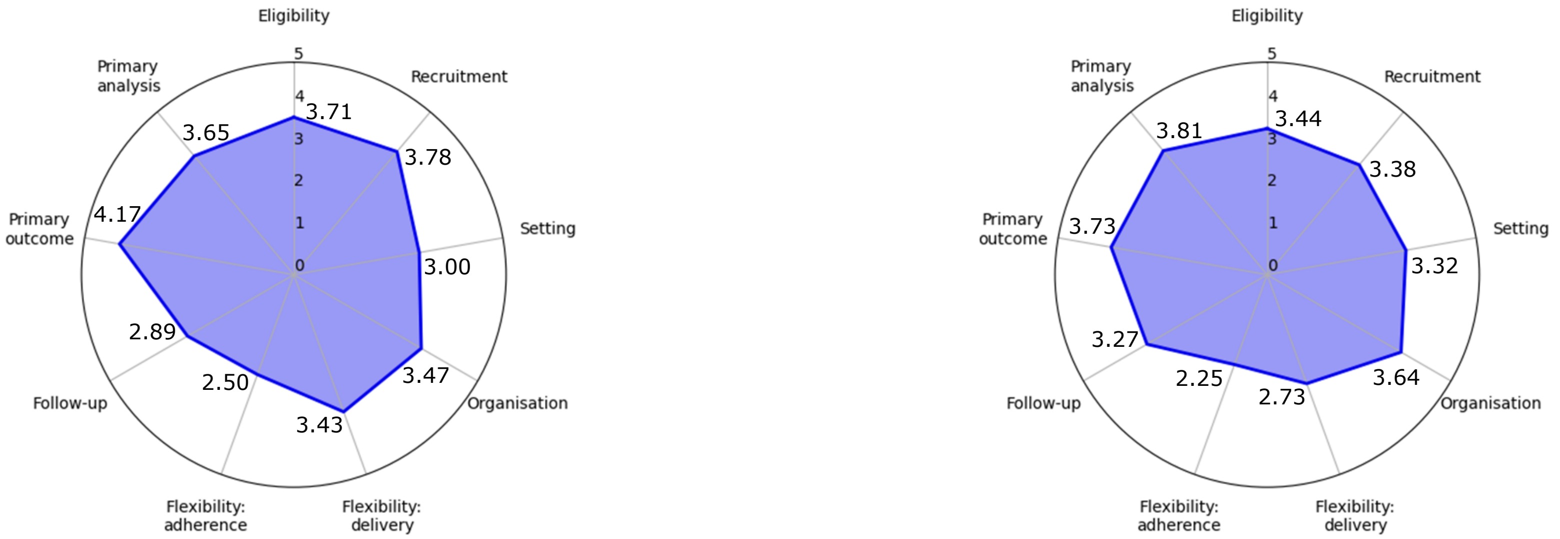

Preliminary(pilot, feasibility) study

Original study

Figure S3. PRECIS-2 score by study type
